# Role of Genetics, Genomics, and Breeding Approaches to Combat Stripe Rust of Wheat

**DOI:** 10.3389/fnut.2020.580715

**Published:** 2020-10-06

**Authors:** Shakra Jamil, Rahil Shahzad, Shakeel Ahmad, Rida Fatima, Rameesha Zahid, Madiha Anwar, Muhammad Zaffar Iqbal, Xiukang Wang

**Affiliations:** ^1^Agricultural Biotechnology Research Institute, Ayub Agricultural Research Institute, Faisalabad, Pakistan; ^2^State Key Laboratory of Rice Biology, China National Rice Research Institute, Hangzhou, China; ^3^Department of Plant Breeding and Genetics, University of Agriculture, Faisalabad, Pakistan; ^4^College of Life Sciences, Yan'an University, Yan'an, China

**Keywords:** fungal pathogen, *Puccina striiformis*, yellow rust, wheat, resistance genes, new breeding strategies

## Abstract

*Puccinia striiformis* (Pst) is a devastating biotrophic fungal pathogen that causes wheat stripe rust. It usually loves cool and moist places and can cause 100% crop yield losses in a single field when ideal conditions for disease incidence prevails. Billions of dollars are lost due to fungicide application to reduce stripe rust damage worldwide. *Pst* is a macrocyclic, heteroecious fungus that requires primary (wheat or grasses) as well as secondary host (*Berberis* or *Mahonia* spp.) for completion of life cycle. In this review, we have summarized the knowledge about pathogen life cycle, genes responsible for stripe rust resistance, and susceptibility in wheat. In the end, we discussed the importance of conventional and modern breeding tools for the development of *Pst*-resistant wheat varieties. According to our findings, genetic engineering and genome editing are less explored tools for the development of *Pst*-resistant wheat varieties; hence, we highlighted the putative use of advanced genome-modifying tools, i.e., base editing and prime editing, for the development of *Pst*-resistant wheat.

## Introduction

**Figure 1:**
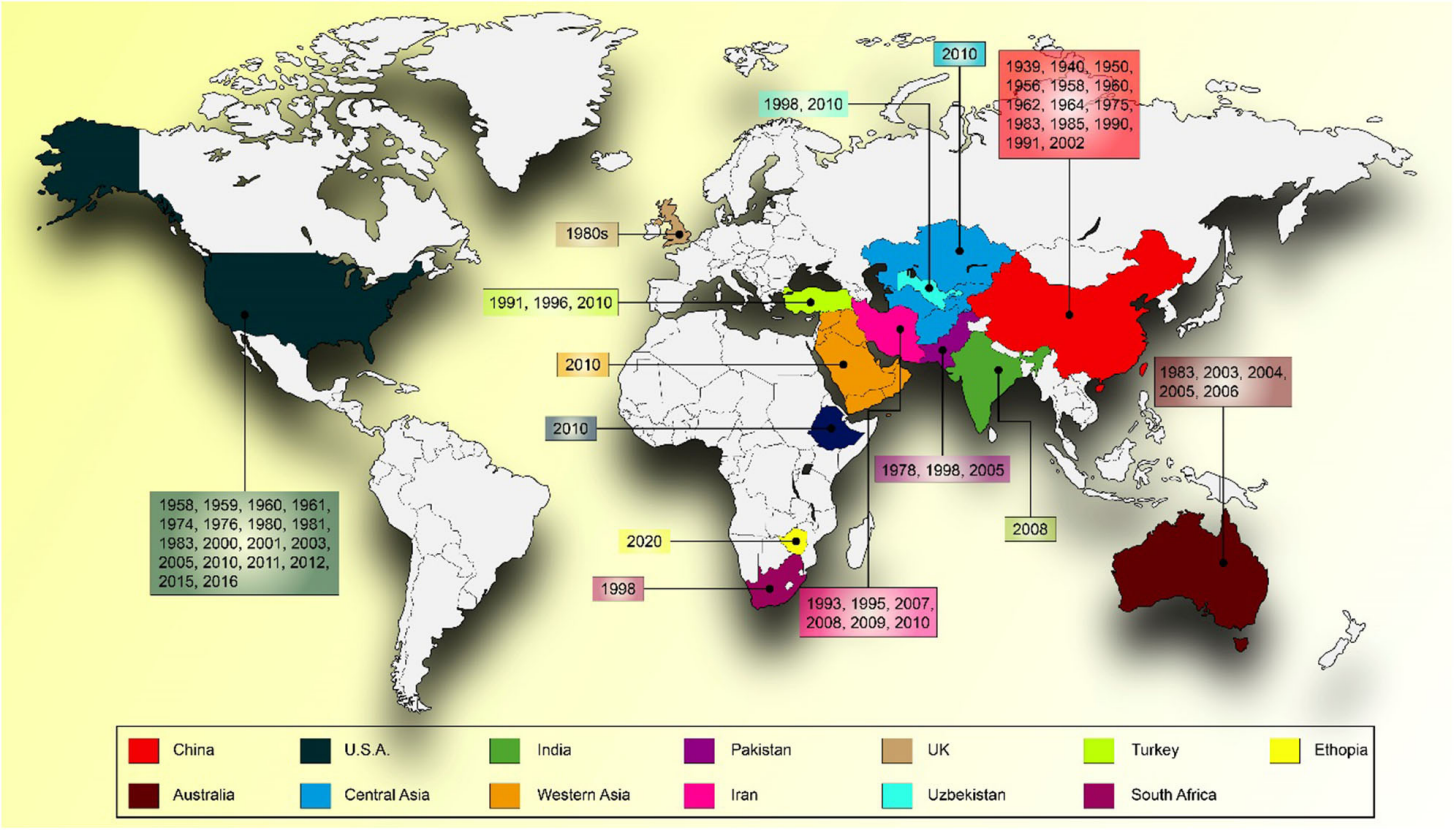
World map showing the epidemic years of stripe rust in different countries around the globe.

Yellow rust caused by *Puccinia striiformis* f. sp. *tritici* (*Pst*) is an economically important disease of wheat. It is also called stripe rust because of the appearance of yellow streaks (pre-pustules), followed by small, bright yellow, elongated uredial pustules arranged in conspicuous rows on the leaves. Stripe rust is an epidemic fungal disease of spring as well as winter wheat. It has been frequently reported from over 60 countries in all continents except Antarctica where wheat is not grown (1). The latest entry to the stripe rust epidemic countries list is Zimbabwe (2). However, the epidemic occurs more frequently (2 or 3 years of every 5 years) in Pakistan, India, China, Nepal, Yemen, Ethiopia, Uzbekistan, Kenya, Australia, the United Kingdom, the United States, Chile, New Zealand, Ecuador, Peru, Colombia, and Mexico (1) (Figure 1).

In the past couple of decades, *Pst* has devastated world wheat production, leaving 80% of wheat varieties susceptible to it. An annual loss of 5 million tons with an estimated cost of ~USD 1 billon is observed every year (3). So far, 51 major stripe rust epidemics have been observed globally from 1939 to 2016, varying in intensity from 2% yield losses to total crop failure (1). Up until now, more than 78 stripe rust resistance genes have been identified that are classified into phenotypically and mechanistically diverse classes, i.e., race-specific (seedling resistance genes) and race-non-specific [adult plant resistance (APR) genes]. Conventional breeding plays an essential role in crop improvement but usually entails growing and examining large populations of crops over multiple generations, a lengthy and labor-intensive process (4), whereas in modern era, gene hunting techniques and understanding about pathogen virulence pattern is increasing by introduction of modern genomics and breeding tools (5). Hence, we have summarized the latest developments in the field of genomics, genetic engineering, and genome editing in comparison to conventional breeding approaches and their role in the development of durable stripe rust-resistant wheat varieties.

Briefly, in this review, we have discussed the recent developments in genetics, genomics, and breeding of rust-resistant wheat varieties. Additionally, we have discussed the modern genomic tools, i.e., genetic engineering, RNAi, and genome editing, in comparison to conventional breeding and mutation breeding and their possible use in the development of *Pst*-resistant wheat varieties.

## The *Pst* Pathogen

The *Pst* is an obligate fungal parasite that uses host plant photosynthetic machinery to fulfill its dietary requirements. *Pst* completes its life cycle in two different and unrelated host species, i.e., wheat and *Berberis* spp. (6). Three out of five different stages in the *Pst* life cycle, i.e., uredial, telial, and basidial phase, are completed on a primary host (wheat), whereas the pycnial and aecial phase needs an alternate host (1). *Pst* has a complex and specialized infection process including spore attachment to host plant, germination, formation of appressorium, and obtaining nutrients by haustorium formation and host invasion. Haustoria are surrounded by special “extrahaustorial membrane” and a gel-like matrix “extrahaustorial matrix” within the living host (7). Urediniospores usually grow when it rains freely with some moisture followed by a temperature of about 7–12°C minimum and 20–26°C maximum. With the passage of time, pathogens have adapted to high temperature, and severe outbreaks are occurring in subtropical areas as well. *Pst* as an airborne pathogen can travel thousands of miles to cause sudden disease epidemics (8).

*Pst* is evolving its race at rapid speed and is becoming a giant (9). However, race evolution and pathogenicity behavior are less explored. These unsolved issues provoked scientists and diverted their attention toward sequencing of the *Pst* genome to provide a deep insight into pathogenicity and host–pathogen interaction (10). To date, more than 15 *Pst* reference genomes are publicly available (11). Bioinformatics predictions have indicated that over 1,000 effector proteins exist in the *Pst* genome; however, their specific role during the infection process is yet to be explored. Advancements in the field of bioinformatics and genomics will help understand the pathogenicity process and race evolution of *Pst* (12). Draft of wheat genome is also available (13), which should also be explored for NBS-LRR and other gene families using bioinformatics tools to figure out putative *Pst* resistance genes for the development of durable *Pst-*resistant wheat cultivars (14).

## Genetics and Genomics of Wheat to Combat *Pst*

Many efforts are directed toward isolating rust resistance (R) genes in crop plants and understanding how to best deploy them for durable resistance. R genes are divided into two mechanistically and phenotypically diverse classes, i.e., seedling and APR genes. R genes encode immuno-receptors in plants to recognize specific pathogen effector proteins and start immune response to stop activity initiated by pathogens through hypersensitive response (15). Effectors are an array of proteins secreted by pathogens to target plant signaling and block immunity or may give rise to effector-triggered immunity (16).

In contrast to R genes, there are also susceptibility (S) genes in plants that promote and facilitate the proliferation of any disease or pathogen attack. These genes negatively regulate the plant resistance against disease and usually include sugar transporters, i.e., SWEET gene family that transport sugar out of the cell and made available to pathogens (17). Similarly, some members of the NAC gene family are also used as target by the host for their multiplication (18). Thus, durable and broad-spectrum disease resistance to *Pst* can be acquired by manipulating the S gene/s, i.e., *TaSTP13*, via the gene modification technique such as the clustered regularly interspaced short palindromic repeats (CRISPR)-associated protein (Cas) system. However, for a better understanding of wheat genetics and genomics for combating *Pst*, both R and S genes have been discussed below.

### R Genes

Seedling resistance, also known as qualitative, vertical, or all stage resistance (ASR), counters one or a few *Pst* races. Seedling resistance genes (SRGs) express at seedling as well as adult plant stages and show phenotypes of major influence (5). SRGs are characterized by a strong to moderate immune response that fully halts pathogen sporulation and disease development. Majority of the SRGs encode nucleotide-binding site leucine-rich repeat (*NBS-LRR*) R proteins that identify effector proteins inside cytoplasm secreted by host and initiate defense response to curtail the growth of pathogens. However, variety becomes susceptible after a few years of release because of the evolution of new virulent dominant races (12). SRGs are easy to incorporate in any breeding programs and provide high-level resistance to *Pst* but are not durable. Many historic epidemics were observed in the past due to breakdown of resistance of SRGs, i.e., *Yr2, Yr9, Yr17*, and *Yr27* (1, 19). A novel SRG, *YrSP*, was recently reported, which is a truncated variant of *Yr5*. *Yr5, Yr7*, and *YrSP* all belong to gene cluster on 2B encoding nucleotide-binding and leucine-rich repeat proteins (NLRs) with a non-canonical N-terminal zinc-finger BED domain (20).

APR, also known as quantitative resistance, combats more than one race of the pathogen. APR is race-nonspecific and is a durable type of resistance due to its polygenic nature (21), with each gene having partial influence. Characteristically, APR genes are slow rusting, have a long latent period, and have less and small-sized uredinia formed in 14 days post-inoculation as compared to susceptible wheat plants (22). APR genes slow down the fungal life cycle with reduced sporulation, fungal population size reduction, and loss of genetic diversity. APR is molecularly independent of NBS-LRR proteins as observed in the case of *Yr18* and *Yr46* that encode transporters. Similarly, *Yr36* activates chloroplast-localized kinase, which activates ROS. Currently, the combination of classical R genes with non-classical R genes seems to be an effective strategy to combat *Pst* (12).

High-temperature adult-plant (HTAP) resistance functions under high temperatures at adult stages and is governed by quantitative trait loci (QTLs). Adequate levels of resistance are obtained by three or more genes because individual APR genes provide low levels of resistance (12). The most commonly used APR genes are *Yr9, Yr17*, and *Yr18* (23). Genome-wide association studies (GWAS) identified a new APR resistance gene, *Yr78* (QYr.ucw-6B located on chromosome 6B) (24). Similarly, suppressive subtractive hybridization (SSH) is also a useful approach for the isolation of APR genes (25). Cloning of numerous wheat adult plant resistant genes has given insight into the mechanism of minor gene resistance. A gene, *Yr36*, codes for a chloroplast-localized protein with wheat kinase and START (WKS) lipid binding domains, projected to less reactive oxygen detoxification by phosphorylation of ascorbate peroxidase as the resulting resistance increases (26). The details of SRG and APRG genes identified so far are summarized in Table 1.

**Table 1:** List of seedling and adult plant yellow rust resistance and susceptibility genes with their source species, location on chromosomes, and germplasm resources.

**Gene name**	**Source**	**Resistance type**	**Loc**	**Germplasm resource**
*Yr1*	*T. aestivum*	ASR	2AL	AvSYr1NIL, Chinese 166, Corin, Dalee
*Yr2*	*T. aestivum*	ASR	7B	Derius, Flevina, Hana, HD2329,Heines VII
*Yr3*	*T. aestivum*	ASR	ND	Enkoy, Minister; Vilmorin 23, Staring
*Yr3-a*	*T. aestivum*	ASR	1B	Druchamp, Stephens, Nord Despre
*Yr3-b*	*T. aestivum*	ASR	ND	Hybrid 46
*Yr3-c*	*T. aestivum*	ASR	1B	Minister, Maris Beacon
*Yr4*	*T. aestivum*	ASR	3BS	Avalon, Bolac, EMU S, Nesser, Hybrid 46
*Yr4-a*	*T. aestivum*	ASR	6B	Vilmorin 23, Yamhill, Cappelle-DespreZ
*Yr4-b*	*T. aestivum*	ASR	6B	Hybrid 46
*Yr5*	*T. spelta*	ASR	2BL	AvSYr5NIL, By33, E5557, E8510
*Yr6*	*T. aestivum*	ASR	7BS	Fielder, Heines Klben, Koga II, Recital, Takari
*Yr7*	*T. aestivum*	ASR	2BL	Brock, Lee, PBW12, Talent, Tango, Tommy
*Yr8*	*Aegilops comosa*	ASR	2A/D	Compair, Hobbit Sib, Maris Widgeon
*Yr9*	*Secale cereale*	ASR	1BS	AvsYr9NIL, Clement, Petkus, Kavkaz
*Yr10*	*T. aestivum*	ASR	1BS	AvSYr10NIL, Moro, PI178383
*Yr11*	*T. aestivum*	APR	ND	Joss Cambier, Heines VII
*Yr12*	*T. aestivum*	APR	ND	Fleurus, Frontier, Pride, Mega
*Yr13*	*T. aestivum*	APR	ND	Maris Huntsman, Hustler
*Yr14*	*T. aestivum*	APR	ND	Kador, Hobbbit, Maris Bilbo, Score, Wembley
*Yr15*	*T. dicoccoides*	ASR	1BS	AvSYr15NIL, Agrestis, Boston, Cortez
*Yr16*	*T. aestivum*	APR	2DS	Bersee, Cappelle-Desprez
*Yr17*	*T. ventricosum*	ASR	2AS	AvSYr17NIL, Apache, Arche, Balthazar, Bill
*Yr18*	*T. aestivum*	APR	7DS	Jupateco, 73R, Wheaton, Opata, Anza, Chris
*Yr19*	*T. aestivum*	ASR	5B	Compare
*Yr20*	*T. aestivum*	ASR	6D	Fielder
*Yr21*	*T. aestivum*	ASR	1B	Lemhi
*Yr22*	*T. aestivum*	ASR	4D	Lee
*Yr23*	*T. aestivum*	ASR	6D	Lee
*Yr24*	*T. turgidum*	ASR	1BS	AvSYr24NIL, Chuanmai 42
*Yr25*	*T. aestivum*	ASR	1D	Carina, Heine VII, Hugenout, TP981, Tugela
*Yr26*	*T. turgidum*	ASR	1BL	Nei 2938, Nei 4221, Yangmai 5
*Yr27*	*T. aestivum*	ASR	2BS	Ciano 79, Inquilab 91, Kauz, Opata 85, Crow
*Yr28*	*Ae. tauschii*	ASR	4DS	Altar 84/Ae. Tauschii W-219, Synthetic
*Yr29*	*T. aestivum*	APR	1BL	Lalbahadur (Parula 1B), Attila, Pavon F76
*Yr30*	*T. aestivum*	APR	3BS	Opata 85, Parula, Inia 66, Pavon F76, Quaiu 3
*Yr31*	*T. aestivum*	ASR	2BS	Pastor
*Yr32*	*T. aestivum*	ASR	2AL	Anouska, Caribo, Consort, Cyrano, Danis
*Yr33*	*T. aestivum*	ASR	7DL	Batavia, EGA Gregory, Strezeck
*Yr34*	*T. aestivum*	ASR	5AL	US22857, WAWHT2046=AUS91389
*Yr35*	*T. dicoccoides*	ASR	6BS	98M71=AUS 91388=T. dicoccoides479/7*CS
*Yr36*	*T. dicoccoides*	HTP	6BS	Glupro, RSL65, Burnside, Lilian, Farnum
*Yr37*	*Ae. kotschyi*	ASR	2DL	Line S14, Line 8078
*Yr38*	*Ae. sharonensis*	ASR	6A	Line 0352-4
*Yr39*	*T. aestivum*	HTP	7BL	Alpowa
*Yr40*	*Ae. geniculata*	ASR	5DS	TA5602, TA5603
*Yr41*	*Triticum aestivum*	ASR	2BS	AIM5, AIM6, Chuannong 19
*Yr42*	*Ae. neglecta*	ASR	6A	Line 03M119-71A
*Yr43*	*T. aestivum*	ASR	2BL	IDO337s=PI 591045, Lolo
*Yr44*	*T. aestivum*	ASR	2BL	Zak=PI 607839
*Yr45*	*T. aestivum*	ASR	EDL	PI 181434, PI 660056
*Yr46*	*T. aestivum*	ASR	4DL	PI 250413, RL6077 = Thatcher*6/PI 250413
*Yr47*	*T. aestivum*	ASR	5BS	AUS28183=V336, AUS28187
*Yr48*	*Synthetic wheat*	APR	5AL	RIL4 GSTR 13504 and RIL167, GSTR 1361
*Yr49*	*T. aestivum*	APR	3DS	Avocet S*3/Chuanmai 18, Chuanmai 18
*Yr50*	*Thi. intermedium*	ASR	4BL	CH223, TAI7047
*Yr51*	*T. aestivum*	ASR	4AL	Line 5515, AUS 91456, AUS2785
*Yr52*	*T. aestivum*	HTP	7BL	PI 182527, PI 660057
*Yr53*	*T. turgidum*	ASR	2BL	PI 480148, PI 67959
*Yr54*	*T. aestivum*	APR	2DL	Yr54 RIL GID6032334, Quaiu 3
*Yr55*	*T. aestivum*	APR	2DL	Frelon AUS38882
*Yr56*	*Durum wheat*	ASR	2AS	AUS 91575, Wollaroi = AUS99174
*Yr57*	*T. aestivum*	ASR	3BS	AUS 91463, AUS 27858
*Yr58*	*T. aestivum*	HTP	3BS	W195
*Yr59*	*T. aestivum*	HTP	7BL	PI 178759, PI 660061
*Yr60*	*T. aestivum*	ASR	4AL	Almop
*Yr61*	*T. aestivum*	ASR	7AS	Pindong 34
*Yr62*	*T. aestivum*	HTP	4BL	PI 192252, PI 660060
*Yr63*	*T. aestivum*	ASR	7BS	AUS 27955
*Yr64*	*Durum wheat*	ASR	1BS	PI 331260, PI 660064
*Yr65*	*Durum wheat*	ASR	1BS	PI 480016; PI 679621
*Yr66*	*T. aestivum*	ASR	3DS	AGG91584WHEA = MSP4543.1, VL892
*Yr67*	*T. aestivum*	ASR	7BL	C591, C306, Zhongzhi 1, AGG91586WHE
*Yr68*	*T. aestivum*	APR	4BL	AGG91587WHEA
*Yr69*	*Thi. ponticum*	ASR	2AS	CH7086
*Yr70*	*Ae. umbellulata*	ASR	5DS	L 393-4, WH890
*Yr71*	*T. aestivum*	APR	3DL	Sunco
*Yr72*	*T. aestivum*	ASR	2BL	AUS27506, AUS27894
*Yr73*	*T. aestivum*	ASR	3DL	Teal, Avocet R
*Yr74*	*T. aestivum*	ASR	5BL	Avocet S, Avocet R
*Yr75*	*T. aestivum*	APR	7AL	Ax
*Yr76*	*Durum wheat*	ASR	3AS	Tyee, ARS-Amber, Cara, Chukar, Hyak
*Yr77*	*T. aestivum*	APR	6DS	PI 322118
*Yr78*	*T. aestivum*	APR	6BS	PI 519805

*Loc, location of chromosome; ASR, all stage resistance; APR, adult plant resistance; HTP, high-temperature adult plant resistance; ND, no data*.

Transcriptional factors (TFs) have gained significant importance during the past couple of decades due to their multiple stress responsive behavior. TFs contribute to *Pst* resistance by activation and repression of genes involved in various defense-associated metabolic pathways (27). Overexpression of *TaWRKY62* provides high-temperature seedling-plant resistance to *Pst*. Interestingly, it was observed that switching on *TaWRKY62* activated salicylic acid (SA)- and jasmonic acid (JA)-responsive genes *TaPR1.1* and *TaAOS*, as well as ROS-associated genes *TaCAT* and *TaPOD*. On the other hand, ethylene (ET)-responsive gene *TaPIE1* was downregulated (28). Similarly, *TaLHY* (a MYB TFs) reduces the negative impacts of the *Pst* infection by overexpressing in leaf blade and sheath (29).

*TabZIP74* was activated in response to wounding due to *Pst* and starts resisting it. Silencing of *TabZIP74* increases the susceptibility of wheat to *Pst* (30). *TaNAC4* was overexpressed in response to *Pst* and by exogenously applied methyl Jasmonate (MeJA), ABA, and ethylene, indicating disease control through plant hormones (31). Above 50 TFs, families with thousands of genes have been reported in plants (http://planttfdb.cbi.pku.edu.cn) that play a direct and indirect role in stress tolerance and resistance. The role of majority of TFs is still unknown and needs further exploration.

### S Genes

**Figure 2:**
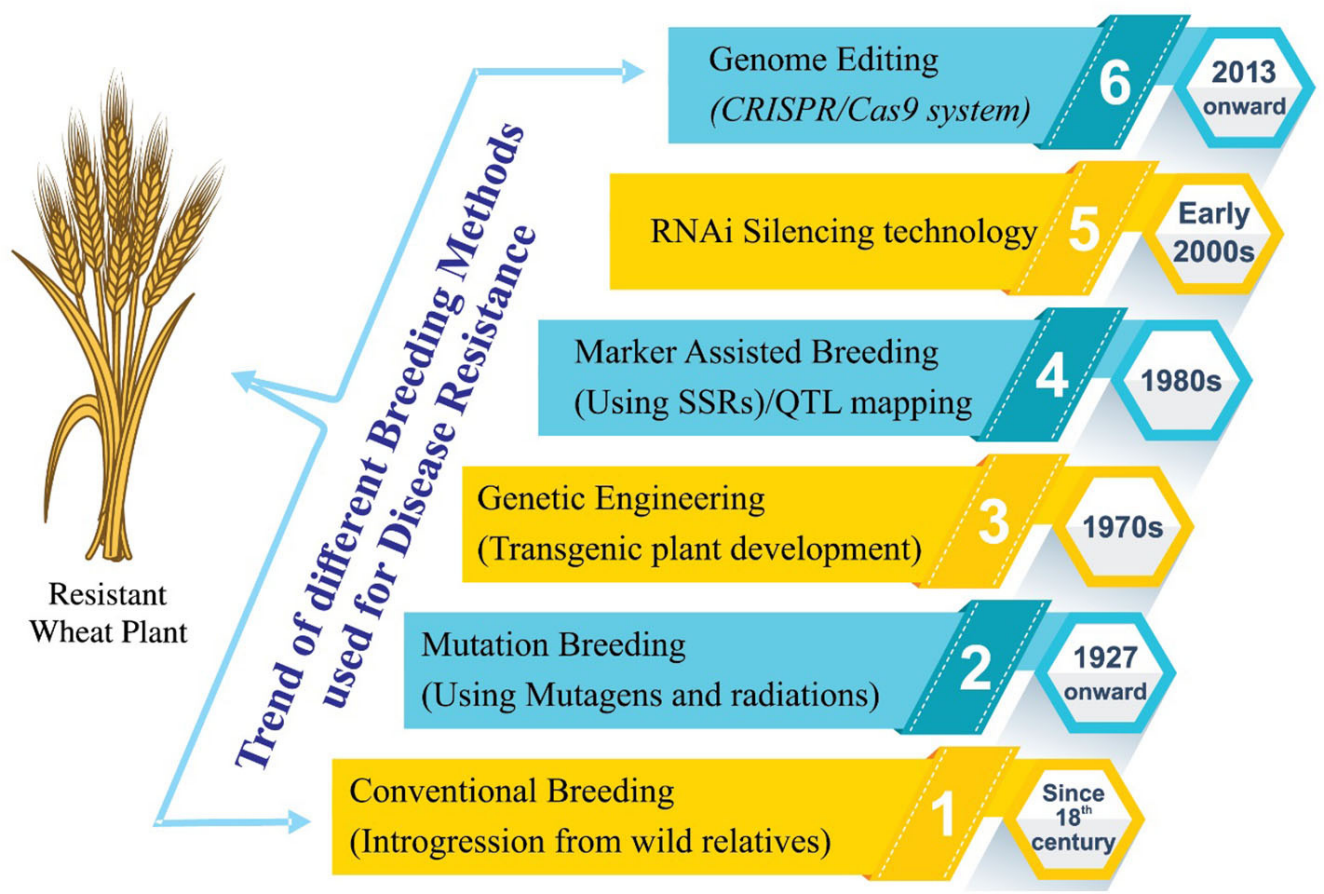
Historical view of different breeding techniques/procedures used for the development of stripe rust-resistant wheat cultivars. Before the advent of mutation breeding in the first quarter of the nineteenth century, conventional breeding approaches were used for resistance incorporation. Later on, advancement in the breeding approaches has brought us to the era of genome editing. Conventional breeding uses conservative breeding tools for the improvement of the trait of interest. Mutation breeding uses physical/chemical mutagens to introduce variation in a population followed by selection. Genetic engineering utilizes recombinant DNA technology for the alteration of the genetic makeup of plants and when the transfer of gene of interest is required from the distantly related organisms. Marker-assisted breeding uses DNA markers for selection of genes of interest and has the advantage of selecting the desirable plants using seedling or even seed sometimes. Genome editing is a way of making specific changes to the DNA of a cell or organism. An enzyme cuts the DNA at a specific sequence, and when this is repaired by the cell, a change or “edit” is made to the sequence.

On the other hand, S genes help pathogens in spreading disease, so by disrupting those genes, plant health can be improved (Figure 2). For example, knocking out *TaSTP13* by barley stripe mosaic virus-induced gene silencing (VIGS) reduced wheat susceptibility to *Pst*. However, its overexpression in Arabidopsis enhanced susceptibility to powdery mildew through increased glucose production in the leaves. These results indicated that *TaSTP13* is transcriptionally induced and contributes to wheat susceptibility to *Pst* by promoting cytoplasmic hexose accumulation for fungal sugar acquisition in wheat–*Pst* interactions (32). It was observed that *Pst* initiated ABA biosynthesis in wheat cells and upregulated *TaSTP6* expression, which increases sugar supply and promotes fungal infection (33). *TaWRKY49* inhibits the expression of *Pst*-responsive genes, and its expression increases when Pst attacks wheat plants (28). Similarly, expression of *TaNAC30* increases in wheat plants attacked by a virulent race (CYR31) of the *Pst*. Knocking out of the *TaNAC30* by VIGS reduced colonization of the virulent *Pst* isolate CYR31. Detailed histological analyses indicated that *TaNAC30* restricts the accumulation of H_2_O_2_ and promotes disease development (18).

## Applications of Advanced Genomics Tools for Identification of Rust Resistance Genes

Advanced genomics tools such as GWAS and QTL-Seq are paving the way for fast-track genetic mapping of traits in various crops (34). GWAS is an observational study of a genome-wide set of genetic variants in different individuals to see if any variant is associated with a trait of interest (35), whereas QTL-Seq combines bulked segregant analysis (BSA) and high-throughput whole-genome re-sequencing to detect the major locus of a certain quantitative trait in a segregating population (36). GWAS was extensively used for searching of novel stripe rust resistance genes as described in the following examples. Two novel stripe rust resistance QTLs were identified using the GWAS approach on 5AS and 5AL wheat chromosomes (37). Similarly, many other GWAS-based studies have identified stripe rust resistance genes/QTLs (38–40). Keeping in view these highlighted examples for the use of GWAS and QTL-Seq in the identification of novel stripe rust resistance genes, both these approaches should be given primary importance in breeding programs focused on development of rust-resistant wheat varieties.

## Breeding Strategies to Develop *Pst*-Resistant Wheat Varieties

A detailed breeding strategy has been proposed for effective control of stripe rust resistance in wheat. A pictorial history of the below-mentioned breeding methodologies is given in Figure 2.

### Conventional Breeding

Conventional breeding approaches include, e.g., selection (pure line selection, mass selection), introduction, hybridization, wild hybridization, backcrossing, composite crossing, multi-line breeding, polyploidy, and heterosis breeding. Hybridization is followed by different breeding methods for the achievement of homozygosity in the filial generation such as pedigree method, bulk, single seed descent (SSD), ear to row, and many other methods that take 8–10 years for development and release of commercial cultivars for field testing as described briefly by Breseghello et al. (41). Phenotyping is done through visual observations under natural field conditions. Selection merely on phenotypic basis under natural infestation does not give satisfactory results and often leads toward selection of false-positive plants. Selection efficiency, however, can be improved by use of artificial inoculation. Still, this is a time-consuming and laborious task with underwhelming results. However, potential exists in wild hybridization. A detailed history of varieties developed through conventional breeding for *Pst* resistance is given in Table 1.

### Marker-Assisted Breeding (MAB)

At the end of the twentieth century, *MAB* is increasingly used in different crop breeding programs with many advantages as compared to conventional breeding. Markers are selected on the basis of their linkage with gene of interest (*GOI*) (42, 43). *MAB* is frequently used for the development of disease-resistant varieties by probing the desirable markers (44). About 78 *Pst* resistance genes had been reported, many of which are race-specific in nature. A single resistant gene may become rapidly susceptible to new fungal races, causing breakdown of resistance; hence, gene pyramiding through *MAB* is a rational approach (5, 8). Stacking of multiple APR *Pst* resistance genes through *MAB* will result in durable stripe rust-resistant wheat genotypes (45). DNA markers are reported for many *Pst* resistance genes, i.e., *Yr5, Yr9, Yr15, Yr17, Yr26, Yr33*, and *YrH52*, as available on MAS wheat (https://maswheat.ucdavis.edu/) along with their complete protocol for *MAB*. *SSR* markers are widely used for *Pst* resistance gene pyramiding due to its highly polymorphic and co-dominant nature (46). Likewise, *SNP* markers using gene-chip technology provides a better-quality approach regarding *Pst*-resistant *QTL* mapping (47).

### Mutation Breeding

Mutation increases biodiversity and also helps conventional plant breeding through the creation of novel variants that do not occur in nature (48). Continuous decline in genetic diversity has paved the way toward mutation breeding (49). Physical (irradiation) and chemical (alkylating agents, nitrous acid, base analogs, etc.) mutagens are used for the induction of mutation in plants. The molecular techniques of DNA fingerprinting, i.e., Random Amplified Polymorphic DNA (RAPD), Amplified Fragment Length Polymorphisms (AFLP), and Sequence-Tagged Microsatellite Sites (STMs), have helped in screening and analysis of desired mutants (50). Mutation breeding through physical or chemical means helps in the development of new varieties with exploitation of more genetic variability. In the present era, mutation breeding is important for modern plant breeding, recombination breeding, and transgenic breeding side by side as detailed in the following examples.

Chemical mutation was induced in NN-Gandum-1, a wheat variety with 0.55% absolute resistance to yellow rust. This mutagenesis resulted in substitution of glutamic acid with alanine through SNP, which helped to alter protein structure. The maximum number of SNPs was found on chromosome 2B, and the minimum was found on chromosome 7D (51). A wheat mutant R39 showed APR to stripe rust, which was identified using specific length amplified fragment (SLAF) sequencing combined with bulk segregant analysis (BSA) and next-generation sequencing (NGS) (52). Gamma rays and electron beam (EBM) irradiation were used in two varieties, PBW343 and HD2967. Doses used were 250, 300, and 350 Gy and 150, 200, and 250 Gy of gamma rays and EBM, respectively. The EBM-derived population showed more mutants compared to the gamma ray population. Absence of sporulation indicates that resistance has been incorporated through mutation breeding (53).

### Genetic Engineering

Genetic engineering is a novel plant breeding tool that involves production of transgenic plants by transformation of GOI in order to obtain desired results (54). Genetic engineering has wider applications for development of biotic and abiotic stress-tolerant crop plants and improving their nutritional quality (55). Genetic engineering has played a significant role in the development of fungal-resistant plants by producing pathogenesis-related protein, i.e., chitinase and β**-**1,3-glucanase. When fungal pathogens attack GM plants, chitinase is produced in large amounts to reduce the hyphal growth (56) as chitinase hydrolyzes fungal cell wall (57). Rice class I chitinase *OsRC24* gene was transformed to genetic background of wheat for the development of *Pst-*resistant wheat plants. The *OsRC24* possessing lines exhibited 27–36% more yield (58).

Similarly, transgenesis of barley *chi26* gene in bread wheat by using biolistic bombardment was also effective in resisting *Pst* (57). Transformation of another gene, *EuCHIT1*, from *Eucommia ulmoides* in wheat had shown enhanced resistance to *Pst* (59). *YrU1* was transformed to wheat from its progenitor *Triticum urartu* and transformed lines showed enhanced resistance against *Pst* (60). Sometimes, gene transcription becomes silent in transgenic plant families, which results in very limited or no expression (57). However, overall results of transgenic technology have shown good promise against rust infestation in wheat. In the future, genetic transformation could be used as a rapid source to obtain *Pst* wheat cultivars and minimize yield losses.

### RNAi Silencing

RNA interference (RNAi) is a conserved mechanism in eukaryotic organisms with a very crucial role in defense mechanism against pathogenic infections and gene regulation (61). RNAi alters the gene function or silencing of crucial pathogenic genes of the pathogen. In order to silence any gene, RNAi uses double-stranded RNA (dsRNA), which is homologous to GOI (62). The natural pathway for RNAi silencing is cleaving double-stranded RNA (dsRNA) into 21- to 26-nucleotide-long small RNA (sRNA), which are short interfering RNAs (siRNAs) or microRNAs (miRNAs). These sRNAs are involved in various processes, i.e., maintaining RNA stability, processing, and response to various biotic stresses (63).

Pathogenesis-related genes in *Pst* were silenced using Barley stripe mosaic virus (BSMV) as a vector for expression of dsRNA homologous to the *Pst* target gene. The MAPK kinase *PsFUZ7* gene, which is an important pathogenicity factor of *Pst* causing fungal infection and regulating hyphal morphology and development on host plant, was knocked down using RNAi. The RNAi constructs targeting the *PsFUZ7* gene of *Pst* was successfully expressed in transgenic wheat lines and conferred strong and durable resistance (62). Similarly, the *PsCPK1* gene (an important transcript of *Pst* expressed at early infection stage) was knocked down in transgenic wheat lines using RNAi (61). Other successful knockouts of *Pst* using transgenic wheat lines are *PsHXT1* (64) and *PstGSRE1* (65) genes, which are a Hexose transporter and an effector protein, respectively. Both these genes are required for pathogenicity. RNAi is an emerging genetic approach with great potential against *Pst*.

### Genome Editing

Plant genome editing uses sequence-specific nucleases (*SSNs*) for stably inherited and predetermined gene modification by introducing favorable alleles into our crop of interest that results in a transgene-free desired genome (66). *SSNs* cause certain changes at the chromosomal level, which results in deletion, insertion, or substitution of specific nucleotide sequence at particular loci. Various types of SSNs, i.e., Zinc finger nucleases (*ZFNs*) and Transcription activator-like effector nucleases (*TALENs*), and the CRISPR*-*Cas system are being used for plant genome editing (67). Targeted genome editing has become the preferred genetic tool for resistance incorporation in plants against various pathogenic diseases (68). Susceptible genes of crop plant are edited and rewritten in such a way to transform them to resistant genes (69).

Nowadays, the CRISPR-Cas9 system, along with its variants, has more applications over other genome editing tools because it is easy to operate, time efficient, and cheap, and has a high success rate (70). For the sake of it, short guided RNA (sgRNA) are designed with which Cas9, an RNA-guided DNA endonuclease, makes a complex for exact targeting of a specific gene (71). In wheat, CRISPR-Cas9 has successfully shown resistance against powdery mildew by making *TaEDR1* mutant wheat plants by simultaneous modification of *TaEDR1* along with its three homologs (72). Similarly, CRISPR-Cas9 was also used for making a resistant, non-transgenic tomato variety against fungal mildew through deletion (67).

**Figure 3:**
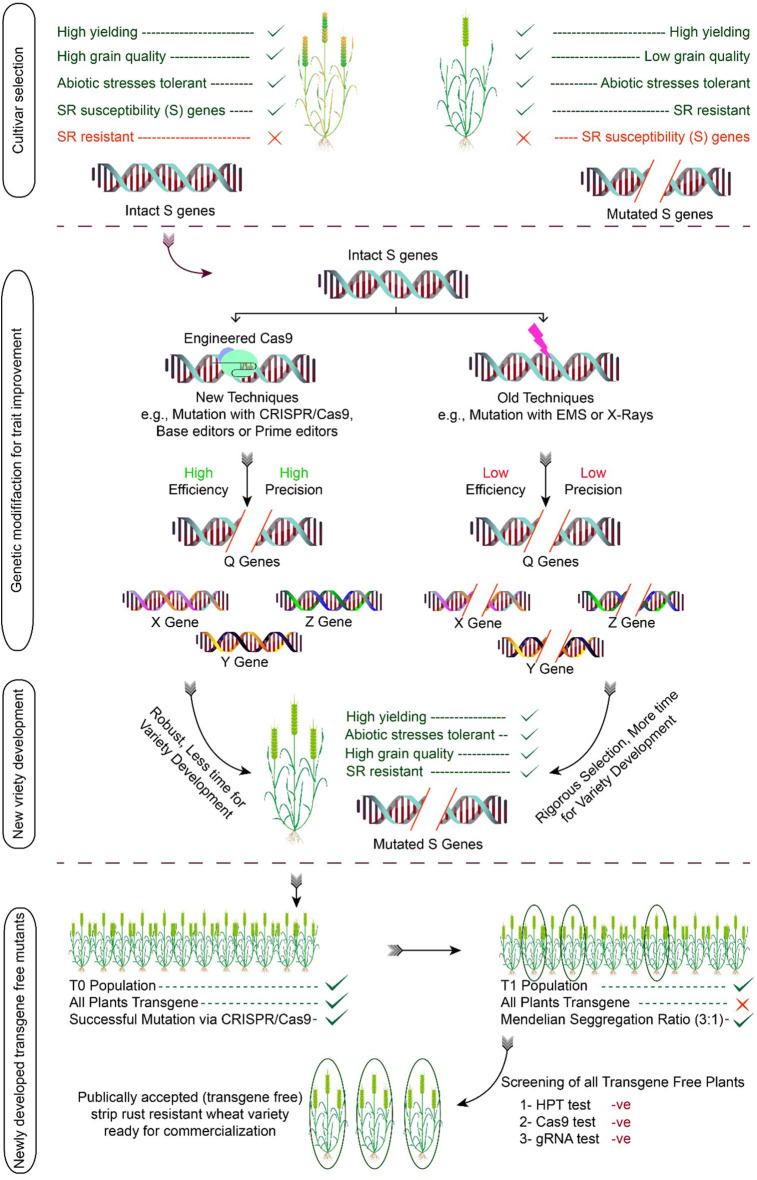
Development of stripe rust-resistant wheat variety using the CRISPR/Cas system. The process begins by selecting the cultivars having the S gene to be modified followed by trait improvement through genome editing. The modified plants are used in the breeding program for the development of variety, and in later generation, transgene-free plants are selected. The illustration also discusses the pros and cons of conventional breeding vs. genome editing. The efficiency of conventional breeding techniques is low as compared to genome editing. Similarly, the off-targeting effects of conventional mutations tools are relatively high in comparison to genome editing.

Another highlighted example of the CRISPR-Cas9 system is the successful editing of three targeted genes, i.e., *TaABCC6, TaNFXL1*, and *TansLTP9*, in the wheat protoplast system for activation of defense mechanism against *Fusarium head blight (FHB)* (73). The potential role of CRISPR-Cas9 in developing *Pst*-resistant plants is yet to be explored. Although different groups are working on the use of CRISPR-Cas9 for developing yellow rust resistance plants, no report has been published yet. However, a great variety of potentials exist for its use. This innovative technique could be used for stripe rust resistance in wheat by predetermined modification of defense genes that will be different from transgenic for increasing wheat production in a time-efficient manner. CRISPR-Cas09 figure in the article after this portion and re-labeled it as Figure 3.

### Speed Breeding

Speed breeding is a novel plant breeding technique that shortens the harvest time of crops by using optimal light intensity, light quality, day length, and controlled temperature for accelerated photosynthesis, flowering, early seed harvest, and shortened generation time. The technology can be used to obtain up to six generations per year of spring and durum wheat, barley, peas, and chickpea and up to four generations of canola under normal greenhouse conditions (74). The technology accelerates plant development in fully enclosed, controlled-environment growth chambers. The light supplementation through LEDs in a glasshouse environment allows rapid generation cycling through SSD and the potential for adaptation to larger-scale crop improvement programs. Unlike double haploid technology, speed breeding does not require specialized labs and can be used for diverse germplasm. The innovations in the LEDs and extended photoperiods coupled with early seed harvest technology have further reduced the generation period (75). Speed breeding was widely exploited in wheat for aboveground (plant height, tolerance to crown rot, and resistance to leaf rust) and belowground (seminal root angle and seminal root number) trait improvement (76, 77). Similarly, it was applied in barley for leaf rust, net blotch, and spot blotch resistance incorporation through modified backcross strategy using multi-trait phenotypic screens (78). These examples highlighted that potential exists for the development of yellow rust-resistant wheat varieties in a shorter time span.

## Conclusion and Future Prospects

The frequent outbreak of *Pst* epidemics and worldwide distribution highlighted the need for an effective control strategy. The pattern of evolution of *Pst*, population structures, migration routes, and reproduction modes are well-known. The advent of genome editing and RNAi silencing technologies has provided a great platform for plant breeders and pathologists to develop resistance varieties. Although breeding resistant cultivars should remain the primary focus, some alternative measures, i.e., integrating fungicides, shifting planting date, changing crop nutrition pattern, eradicating volunteer plants, cultivar mixing, and intercropping, may be adopted as a temporary solution. Side-by-side development and distribution of resistant wheat cultivars to provide cost-effective and environment-friendly solution should be continued. Efforts of breeders and pathologists have borne fruit, and to date, ~78 Yr genes have been identified, some of which are seedlings and others are APR genes (8). Marker systems have been optimized for efficient identification of stripe rust resistance genes, which provide the opportunity for pyramiding of multiple *Pst* resistance genes in a cultivar for durable resistance.

Focus should be diverted toward identification of more *Pst* resistance genes from wild relatives. More than 2,500 members of the NBS-LRR gene family have been reported in wheat, 570 in *Triticum urata*, 842 in *Aegilops tauschii*, 316 in *Brachypodium distachyon*, and 420 in *Hordeum vulgare* (79), and majority of these have potential for disease resistance (80). Use of bioinformatics tools will help to understand the functions of the NBS-LRR gene family for hunting putative *Pst* resistance genes. Sequencing of the *Pst* genome provides the opportunity for functional annotation of genes involved in biology and pathogenicity of *Pst*. This has opened new horizons and provided opportunities to understand virulence variation in pathogens and the mechanism of resistance in hosts. *In silico*-based studies should be devised to identify genes responsible for the pathogenicity of *Pst* and mutations responsible for the development of new races. Simultaneous surveying of wheat genome for durable resistance genes from NBS-LRR or other gene families, i.e., TaLTPs (80), needs to be taken into consideration.

The future of plant breeding efforts for durable resistance against *Pst* should not be limited to conventional breeding approaches; instead, novel knowledge-generating tools, i.e., bioinformatics (81), phenomics (82), advanced genomics technologies (83), and novel genome modification techniques, i.e., CRISPR and RNAi (68), should be combined to evolve durable resistance wheat varieties. Similarly, to shorten the breeding time span, double haploid breeding, shuttle breeding, and speed breeding may be incorporated (74, 75). Currently, there are four classes of CRISPR-Cas-derived genome editing agents, including nucleases, base editors, transposases, and prime editors (84). The latest breakthrough in genome editing, i.e., base editing (the irreversible conversion of a base at the target site without involving donor templates, double-stranded breaks, and dependency on NHEJ and HDR), prime editing [the introduction of indels and all 12 base-to-base conversions without inducing a DNA double-strand break using prime editing guide RNA (pegRNA) that drives the Cas9 endonuclease], and genome editing using rice zygote (which overcomes the problem in delivery of macromolecule to the host cells and tissues and difficulty in transformation and regeneration), has opened new horizons for biotechnologists and plant pathologists (84). In this regard, the identification of new S genes is highly needed. In the future, researchers should focus on the identification of new genes that facilitate the proliferation of strip rust pathogen. Later, those genes can be used to improve plant health against strip rust by using genome-editing tools. Briefly, these cutting-edge advancements have paved the way for the fast-track development of stripe rust-resistant wheat varieties.

## Author Contributions

SJ and RS conceived the idea. RS, RF, RZ, and MA drafted the manuscript. SA prepared illustrations. XW provided the literature and technical assistance. SJ, RS, SA, MZI, and XW reviewed and improved the draft. All authors listed have made a substantial, direct and intellectual contribution to the work, and approved it for publication.

## Conflict of Interest

The authors declare that the research was conducted in the absence of any commercial or financial relationships that could be construed as a potential conflict of interest.
